# Periodontitis-associated metabolite isoleucine impairs intestinal barrier function and exacerbates intestinal inflammatory response by NF-κB signaling

**DOI:** 10.3389/fcimb.2025.1684362

**Published:** 2025-11-28

**Authors:** Xiaoxue Wang, Yilin Luo, Jie Ren, Hezhen Xie, Marco Aoqi Rausch, Xiaohui Rausch-Fan, Fei Hu, Xueyang Zhang

**Affiliations:** 1 Department of Stomatology, The Eighth Affiliated Hospital, Southern Medical University (The First People’s Hospital of Shunde, Foshan), Foshan, China; 2 Clinical Division of Periodontology, Center for Clinical Research, University Clinic of Dentistry, Medical University of Vienna, Vienna, Austria; 3 Stomatological Hospital, School of Stomatology, Southern Medical University, Guangzhou, China

**Keywords:** periodontitis, colitis, isoleucine, intestinal barrier function, intestinal inflammatory response, NF-κB signaling

## Abstract

**Objective:**

Periodontitis-associated metabolite isoleucine (Ile) plays an important role in periodontitis aggravating colitis. However, how Ile exacerbates colitis is largely unknown.

**Methods:**

C57BL/6J mice were used to establish experimental periodontitis and colitis models. Histological alterations of the periodontium and colon were observed by HE staining. The gut barrier function was evaluated by intestinal permeability using FITC-dextran. The expression of tight junctions (ZO-1 and occludin) was detected by immunohistochemical staining or immunofluorescence. The NF-κB signaling pathway was detected using qRT-PCR and Western blot.

**Results:**

Experimental periodontitis and periodontitis-associated metabolite Ile increased the intestinal permeability, downregulated the expression of tight junctions (ZO-1 and occludin), and enhanced the NF-κB signaling pathway of intestinal epithelial cells in dextran sulfate sodium (DSS)–induced colitis mice. Ile downregulated the expression of tight junctions (ZO-1 and occludin) and enhanced the NF-κB signaling pathway in intestinal organoids or IEC-6 cells under inflammatory conditions. IKK-16 (a selective inhibitor of IKKβ that prevents NF-κB activation) rescued excessive inflammatory responses induced by Ile in IEC-6 cells with LPS treatment. In addition, IKK-16 relieved the impairment of intestinal barrier function and inflammatory response induced by Ile in DSS-induced colitis mice.

**Conclusion:**

Our study unraveled that periodontitis contributed to intestinal barrier function damage and inflammation of intestinal epithelial cells by potentiating NF-κB signaling in the context of colitis and that this was associated with periodontitis-associated metabolite Ile.

## Introduction

Periodontitis is a chronic inflammatory disease caused by an imbalance between the local microbiota and host immune response (Su et al., 2023; Slots, 2017). It is well known that periodontitis is closely related to systemic health (Kapila, 2021; Wang et al., 2025). Ulcerative colitis (UC) is one of the two forms of inflammatory bowel disease (IBD) and is characterized by symptoms such as abdominal pain, diarrhea, and the presence of bloody mucus in stools, with a clinical course marked by alternating periods of exacerbation and remission (Yan et al., 2025; Wangchuk et al., 2024). Epidemiological studies have established a close association between periodontitis and UC, and animal experiments further demonstrate that periodontitis exacerbates colitis (Kitamoto et al., 2020b; Wang et al., 2023a; Qian et al., 2022; Bao et al., 2022; Han et al., 2023). However, how periodontitis influences IBD remains to be fully elucidated.

Intestinal barrier dysfunction and dysregulated immune response are two key factors affecting the pathogenesis of IBD (Ungaro et al., 2017; Torres et al., 2017). Intestinal epithelial cells (IECs) play critical roles in regulating intestinal barrier function and immune homeostasis (Peterson and Artis, 2014). For example, IECs constitute barrier surfaces that separate the mucosal immune system from the external environment (Peterson and Artis, 2014). The IECs are interconnected to each other by the tight junction (TJ), which is formed by the zonula occludens (ZO) family (ZO-1, ZO-2, and ZO-3), occludin, members of the claudin family, and the junctional adhesion molecule family (Kaminsky et al., 2021). In addition, IECs also secrete cytokines (TNF-α, IL-1β, and IL-6), which are primarily regulated by the nuclear factor (NF)-κB signaling (Chen et al., 2021). Hence, identifying critical molecules that regulate the barrier and immunoregulatory properties of IECs might aid in the development of new strategies to prevent and treat IBD.

Previous studies have reported that periodontitis contributes to IBD by supplying the gut with both colitogenic oral pathobionts and pathogenic T cells (Kitamoto et al., 2020b, Kitamoto et al., 2020a; Liu et al., 2020). In our previous work (Wang et al., 2023a), we found that isoleucine (Ile) was the metabolite of microbiota related to periodontitis and played an important role in periodontitis aggravating colitis (Wang et al., 2023a). Ile is one kind of essential amino acids and is synthesized in bacteria, plants, and fungi but not in animals (Neinast et al., 2019). Numerous studies report that Ile is critical in physiological functions of the whole body, such as protein metabolism, glucose transportation, fatty acid metabolism, and immunity (Gu et al., 2019; Hale et al., 2004; Zhao et al., 2014). However, excessive accumulation of Ile lead to the occurrence and development of various diseases. For instance, during the progression of Alzheimer's disease, gut microbiota dysbiosis causes an abnormal elevation of Ile, resulting in pathological neuroinflammation and cognitive impairment (Wang et al., 2019). Periodontitis patients have significantly elevated Ile level in saliva or gingival crevicular fluid compared with healthy controls (Baima et al., 2021; Garcia-Villaescusa et al., 2018). However, whether and how Ile influences the barrier function and immunoregulatory properties of IECs during colitis progression are largely unknown.

Here, we found that periodontitis might impair intestinal barrier function and exacerbate intestinal inflammatory response via Ile. Furthermore, we proved that Ile reduced the expression of ZO-1 and occludin and upregulated the expression of pro-inflammatory cytokines (TNF-α, IL-6, and IL-1β) through activating the NF-κB signaling pathway *in vivo* and *in vitro*. Taken together, our findings provide a novel perspective in understanding periodontitis exacerbating colitis and potential intervention targets for the clinical treatment of colitis associated with periodontitis.

## Methods

### Mice model

All mouse experimental procedures and protocols were evaluated and authorized by the Animal Ethics Committee of the Institute of Biological and Medical Engineering, Guangdong Academy of Sciences (approval number: 2020011).

It has been reported that female mice develop periodontitis with a higher progression rate compared with male mice (Lin et al., 2021). Therefore, female mice were used to explore the effect of periodontitis on colitis in this study.

An experimental periodontitis and colitis model was established as previously described (Kitamoto et al., 2020b) with minor modifications. Forty specific pathogen-free (SPF) C57BL/6J mice (wild type, female, 8 weeks, 18–20 g) were maintained in an SPF facility under a 12h light/dark cycle (environmental temperature remained around 23°C and humidity around 55%). After a 7-day acclimation, mice were randomly divided into four groups (*n* = 10 per group), including a control group (Con), an experimental periodontitis group (ligature), a colitis group (dextran sulfate sodium, DSS), and an experimental periodontitis and colitis group (ligature-DSS), respectively. The ligature-induced experimental periodontitis model was generated as previously described (Marchesan et al., 2018). Briefly, a dual-knotted silk (100 μm diameter) was placed between the first and second maxillary molars on contralateral right and left sides under isoflurane anesthesia. Silk ligature remained in place for 25 days in ligature and ligature-DSS mice or 3h in Con and DSS mice. After silk ligature for 18 days, DSS and ligature-DSS mice were treated with 1.5% DSS w/v (MP Biochemicals, California, United States) in drinking water for 5 days followed by regular water for 2 days. Mice in the Con and ligature groups were given regular water.

A DSS-induced mouse model with Ile gavage administration was constructed as previously reported (Wang et al., 2023a). Twenty SPF C57BL/6J mice (wild type, female, 8 weeks, 18–20 g) were randomly divided into four groups (*n* = 5 per group), namely the control group (Con), the Ile gavage group (Ile gavage), the colitis group (DSS), and the Ile gavage and colitis group (Ile gavage-DSS), respectively. Mice in the DSS and Ile gavage-DSS groups were treated with 3.0% DSS for 5 days, followed by an additional 2 days of regular water. Con and Ile gavage mice were given regular water. Mice in the Ile gavage and Ile gavage-DSS groups were orally administered with Ile (50 mg/kg/day) for 7 days. Con and DSS mice were orally administered with PBS as a vehicle control. Weigh the mice before each administration and adjusted the dosage.

A DSS-induced mouse model with Ile gavage and IKK-16 administration was established. Fifteen SPF C57BL/6J mice (wild type, female, 8 weeks, 18–20 g) were randomly divided into three groups (*n* = 5 per group), including the colitis group (DSS), the Ile gavage and colitis group (Ile gavage-DSS), and the Ile gavage and colitis group with IKK-16 treatment (Ile gavage-DSS-IKK-16). All mice were treated with 3.0% DSS for 5 days, followed by an additional 2 days of regular water. Ile gavage-DSS and Ile gavage-DSS-IKK-16 mice were orally administered with Ile (50 mg/kg/day) for 7 days. In the DSS group, mice were orally administered with PBS as a vehicle control. Ile gavage-DSS-IKK-16 mice were intraperitoneally injected with IKK inhibition (IKK-16) (1 mg/kg/day) for 7 days. DSS and Ile gavage-DSS mice were intraperitoneally injected with normal saline as a vehicle control. Weigh the mice before each administration and adjusted the dosage.

During the course of all the animal experiments, mice were monitored for weight loss, stool consistency, and hemoccult. After animal modeling was completed, mice were sacrificed via anesthetic overdose with an injection of 120 mg/kg thiopental sodium. Bilateral maxillary molar or colon tissue was collected for the detection of pathological and inflammatory factors.

### Histology and immunostaining

Maxillae and colonic tissues were rinsed with 1× DPBS and fixed in 10% formalin. The maxillae specimens were decalcified in 0.5 M ethylenediaminetetraacetic acid buffer (Servicebio) for 1 month. Then, the maxillae and colonic tissues were dehydrated, clarified, embedded in paraffin, and cut into 5 µm sections for histopathological assessment and immunostaining. Each specimen was cut into five slices, and each slice was photographed in three fields of vision.

Histopathological analysis of maxillae and colonic tissues was performed as previously described (Ren et al., 2025; Xu et al., 2020). After treatment with xylene and serial dilutions of ethanol, the sections were stained using the Hematoxylin and Eosin Staining Kit (Beyotime) according to the manufacturer’s instructions. After that, histological sections were analyzed using light microscopy.

For immunostaining, the sections were stained as previously reported (Xu et al., 2020). The following antibodies were used: anti-Ki67 (1:250, Cell Signaling Technology), anti-ZO-1 (1:150, Servicebio), and anti-occludin (1:100, Servicebio).

### Evaluation of weight, disease activity index, and the histological activity index

Body weight, occult blood status, and stool consistency were determined daily. The disease activity index (DAI) was used for scoring as previously reported (Murthy et al., 1993). histological activity index (HAI) scores of colonic tissues were assigned in a blind manner by a trained pathologist evaluating the following set of variables (Wang et al., 2023a): the loss of goblet cells (0, normal; 1, small; 2, large; 3, most), crypt gland destruction (0, normal; 1, a small amount of crypt loss; 2, a large amount of crypt loss; 3, extensive crypt loss), and the extent of inflammatory cell infiltration (0, normal; 1, infiltration around crypt glands; 2, infiltration of the mucosal muscle layer; 3, extensive infiltration of the mucosal muscle layer with oedema; 4, infiltration of the submucosal layer). An overall score was obtained by summing the scores assigned to each variable.

### Intestinal permeability assay

FITC-dextran (MW 4 kDa; Sigma) was used to detect the intestinal permeability as previously reported (Furuta et al., 2001). Briefly, subject the mice to a fasting treatment for 4h, followed by oral administration of FITC-dextran (600 mg/g body weight). After that, the mice were fasted for 4h again, and then eye frame blood was collected to determine the content of FITC-dextran using the standard curve of FITC-dextran.

### Unbiased transcriptomic analyses

Unbiased transcriptomic analyses were performed according to our previously published methods (Wang et al., 2023a).

### Western blot

The NF-κB signaling pathway was detected using Western blot as previously reported (Wang et al., 2022). Briefly, 50 μg of total protein extracted using lysis buffer (Beyotime) were added to 1× SDS-denaturing loading buffer, boiled for 5 min, and then subjected to 12% SDS-PAGE. SDS-PAGE was blotted with polyvinylidene difluoride membranes (Millipore), and the blotted membrane was incubated with phospho-NF-κB p65 (Cell Signaling Technology), NF-κB p65 (Cell Signaling Technology), Phospho-IκBα (Cell Signaling Technology), IκBα (Cell Signaling Technology), or β-actin (Proteintech Group) primary antibody and added a goat anti-mouse or anti-rabbit HRP-conjugated antibody. Signals were visualized using the ECL Western Blot Kit (BIO-RAD).

### Cell culture

IEC-6 were obtained from the Eighth Affiliated Hospital of Southern Medical University. All experiments were performed with mycoplasma‐free cells. IEC-6 were cultured in Dulbecco’s modified Eagle’s medium supplemented with 10% fetal bovine serum (Biological Industries) in an atmosphere of 5% CO_2_ at 37°C. Cells were exposed to 1 μg/mL LPS (Sigma) or 10 mM Ile (Solarbio) for two days.

### Culture and treatment of intestinal organoid

Crypts were isolated as previously reported (Wang et al., 2023b). Organoids were treated with 100 ng/mL TNF-α (PEPROTECH) for 24h alone and cocultured with 10 mM Ile for another 24h.

### Quantitative real-time PCR

Total RNA was isolated using the FastPure Cell/Tissue Total RNA Isolation Kit V2 (Vazyme) according to the manufacturer’s instructions. UnionScript First-strand cDNA Synthesis Mix (SENO) was applied to synthesize cDNA, which was used to detect gene expression using primers in **Table 1**. All mRNA levels were normalized against β-actin mRNA. Gene expression was calculated based on the 2^-△△Ct^ method (Livak and Schmittgen, 2001).

**Table 1:** Primers used for qRT-PCR.

Gene	Primer sequences (5′-3′)
Mouse-TNF-α	GGTGCCTATGTCTCAGCCTCTT
	GTGGTTTGTGAGTGTGAGGGTCT
Mouse-IL-6	ATAGTCCTTCCTACCCCAATTTCC
	CTGACCACAGTGAGGAATGTCCAC
Mouse-IL-1β	GAAATGCCACCTTTTGACAGTG
	TGGATGCTCTCATCAGGACAG
Mouse-β-actin	AAGTGTGACGTTGACATCCG
	GATCCACATCTGCTGGAAGG
Rat-TNF-α	AGCATGATCCGAGATGTGGAA
	CAGTAGACAGAAGAGCGTGGTG
Rat -IL-6	TTGCCTTCTTGGGACTGATGT
	ACTGGTCTGTTGTGGGTGGTATC
Rat -IL-1β	CCTCTGTGACTCGTGGGATGAT
	TACCACTTGTTGGCTTATGTTCTGT
Rat-β-actin	CCGTAAAGACCTCTATGCCAACA
	CGGACTCATCGTACTCCTGCTT

### Statistical analyses

All statistical analyses were conducted using Prism 8 (GraphPad Software, San Diego, CA). Statistical tests were performed using the two-tailed Student's t-test, and one- or two-way analysis of variance (ANOVA) followed by the Bonferroni post-hoc test. In the figures, asterisks denote statistical significance (*p < 0.05; **p < 0.01; ***p < 0.001; ****p < 0.0001). The data are shown as mean ± standard deviation (SD).

## Results

### Ligature-induced experimental periodontitis impairs intestinal barrier function and promotes intestinal inflammatory response

**Figure 1:**
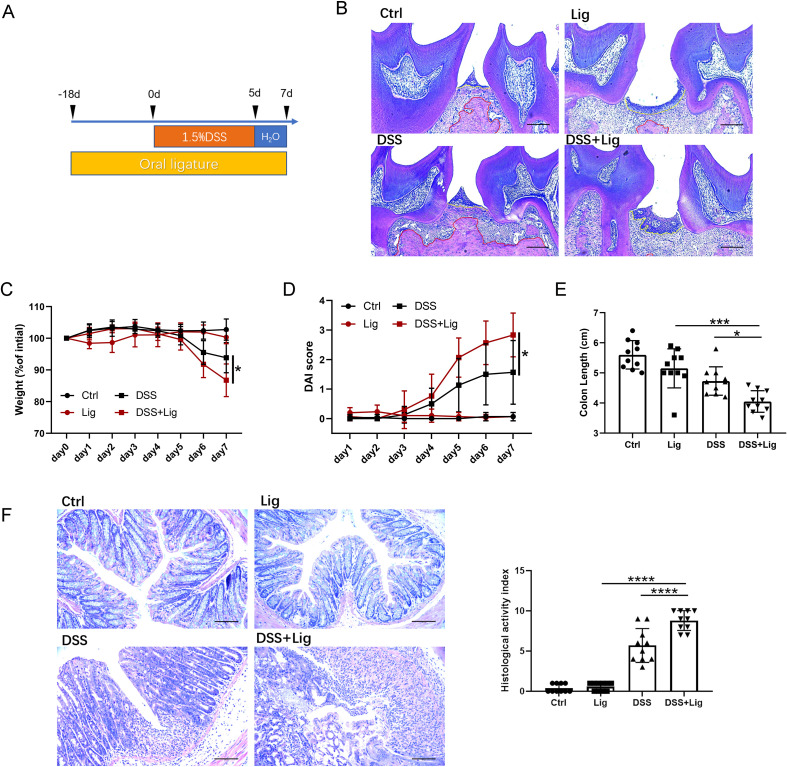
Experimental periodontitis aggravated colitis. **(A)** Schematics of generating a ligature-induced experimental periodontitis and dextran sulfate sodium (DSS)–induced colitis mouse model. **(B)** Representative histological images of gingival tissues (scale bar = 100 μm). The red dash represents the boundary between bone and connective tissue. The yellow dash represents the boundary between connective and epithelium. Statistical analysis of body weight **(C)**, disease activity index (DAI) score **(D)**, and colon length **(E)** in each group. **(F)** Representative colonic histological images and the corresponding histological activity index scores. Each dot indicates an individual mouse (*n* = 10) (scale bar = 100 μm). Results are shown as mean ± standard deviation (SD). *p < 0.05, **p < 0.01, ***p < 0.001, ****p < 0.0001 by two-way analysis of variance (ANOVA) followed by Bonferroni *post-hoc* test **(C, D)** or one-way ANOVA followed by Bonferroni *post-hoc* test **(E, F)**.

A mouse model of periodontitis and colitis was constructed as our previous study did (Wang et al., 2023a) (**Figure 1A**). Infiltration of inflammatory cells, loss of connective tissue attachment, and resorption of alveolar bone were observed in gingiva, indicating that ligature-induced experimental periodontitis was successfully (**Figure 1B**). The weight, DAI values, colon length, and HAI score of mice were monitored. Mice in the ligature-DSS group had significantly increased weight loss, higher DAI values, shorter colon length, and elevated HAI scores in contrast to the mice in the DSS group (**Figures 1C–F**). However, periodontitis alone had no influence on the weight, DAI values, colon length, and HAI score (**Figures 1C–F**).

**Figure 2:**
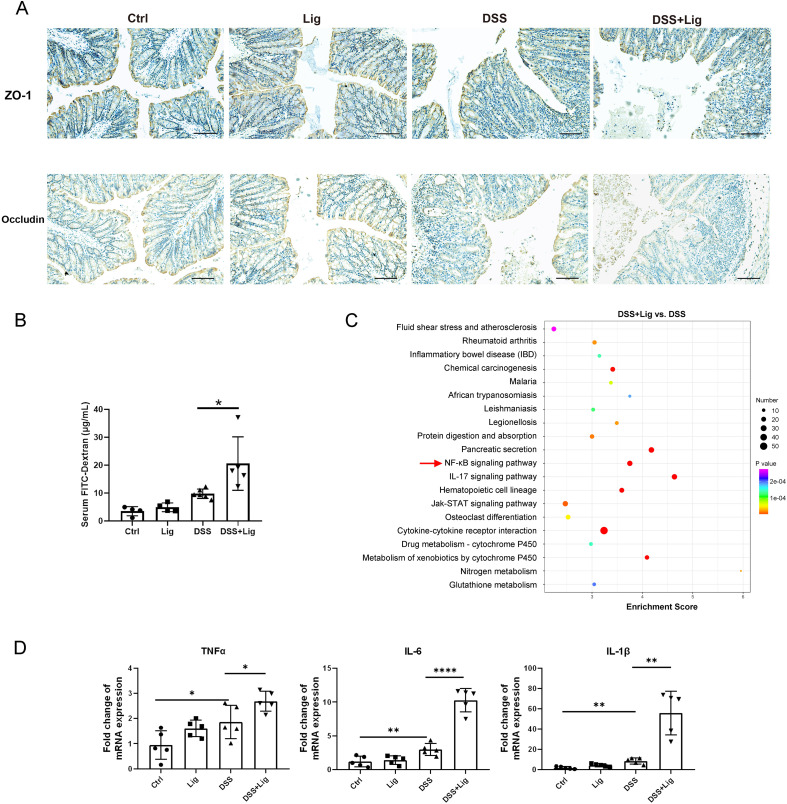
Experimental periodontitis impaired intestinal barrier function and promoted intestinal inflammatory response in mice with DSS-induced colitis. **(A)** Immuno-histochemical staining of ZO-1 and Occludin in colonic sections from each group (scale bar = 100 μm). **(B)** Intestinal permeability was evaluated by measuring the concentration of fluorescein isothiocyanate (FITC)–dextran in serum of each group. **(C)** Kyoto Encyclopedia of Genes and Genomes (KEGG) enrichment analysis of differentially expressed genes in colonic tissue of experimental periodontitis and DSS-induced colitis mice model. **(D)** Quantitative real-time PCR (qRT-PCR) analysis of tumor necrosis factor-alpha (TNF-α), interleukin-1 beta (IL-1β), and interleukin-6 (IL-6) mRNA levels in intestinal epithelial cells (IECs) isolated from the colonic tissue. Each dot indicates an individual mouse (*n* = 5). Results are shown as mean ± SD. *p < 0.05, **p < 0.01, ****p < 0.0001 by one-way ANOVA followed by Bonferroni *post-hoc* test.

To evaluate the effect of periodontitis on intestinal barrier function in colitis mice, immunohistochemical staining of colonic tissue was performed, and the result showed that the expression of TJ proteins (ZO-1 and occludin) was significantly decreased in ligature-DSS mice in contrast to the controls (**Figure 2A**). In addition, we compared the intestinal permeability in our periodontitis and colitis mice model and found that the FITC-dextran concentration of ligature-DSS mice was significantly higher than that of DSS mice (**Figure 2B**), indicating that periodontitis might facilitate intestinal barrier function damage in colitis mice. To investigate the mechanism by which periodontitis impairs the intestinal barrier, we used our previous unbiased transcriptome of the colon tissue (Wang et al., 2023a) to perform KEGG pathway enrichment analysis and found that the NF-κB signaling pathway was markedly enriched in the ligature-DSS group compared with the DSS group (**Figure 2C**). The expression of pro-inflammatory cytokines (TNF-α, IL-6, and IL-1β) downstream of the NF-κB signaling pathway was significantly increased in IECs isolated from the colonic tissue of ligature-DSS mice compared with the controls (**Figure 2D**). However, periodontitis alone had no effect on intestinal barrier function and inflammatory response (**Figures 2A–D**). Taken together, these results indicated that periodontitis impaired intestinal barrier function and aggravated intestinal inflammation of IECs in colitis mice.

### Periodontitis-associated metabolite Ile facilitated intestinal barrier damage and inflammation in the context of colitis

**Figure 3:**
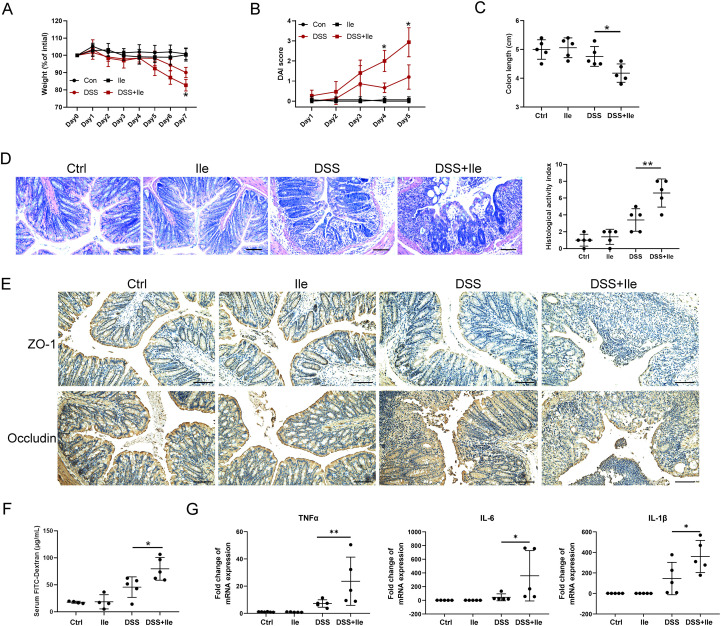
Periodontitis-associated metabolite isoleucine (Ile) facilitated intestinal barrier damage and inflammation in colitis mice. Statistical analysis of body weight **(A)**, DAI score **(B)**, and colon length **(C)** in each group. **(D)** Representative colonic histological images and the corresponding histological activity index scores (scale bar = 100 μm). **(E)** Immuno-histochemical staining of ZO-1 and Occludin in colonic sections from each group (scale bar = 100 μm). **(F)** Intestinal permeability was evaluated by measuring the concentration of FITC-dextran in serum of each group. **(G)** qRT-PCR analysis of pro-inflammatory cytokines (TNF-α, IL-1β, and IL-6) mRNA levels in IECs isolated from the colonic tissue. Each dot indicates an individual mouse (*n* = 5). Results are shown as mean ± SD. *p < 0.05, **p < 0.01 by two-way ANOVA followed by Bonferroni *post-hoc* test **(A, B)** or one-way ANOVA followed by Bonferroni post-hoc test **(C, D, F, G)**.

To explore the influence of Ile on intestinal barrier function in colitis mice, we first established a DSS-induced colitis mouse model with the Ile gavage and found that mice in Ile gavage-DSS group had impaired body weight gain, higher DAI values, shorter colon length, and higher HAI compared with mice in the DSS group (**Figures 3A–D**). Immunohistochemical staining showed that the expression of TJ proteins (ZO-1 and occludin) was significantly decreased in Ile gavage-DSS mice in contrast to the DSS mice (**Figure 3E**). The FITC-dextran concentration of Ile gavage-DSS mice was observably higher than that of DSS mice (**Figure 3F**). Moreover, the expression of pro-inflammatory cytokines (TNF-α, IL-6, and IL-1β) was markedly higher in IECs isolated from the colonic tissue of Ile gavage-DSS mice than that of DSS mice (**Figure 3G**). However, Ile gavage alone had no effect on intestinal barrier function and inflammation (**Figures 3D–G**). These results suggested that periodontitis-associated metabolite Ile promoted intestinal barrier dysfunction and inflammation of IECs in colitis mice.

### Ile aggravated TNF-α–induced intestinal epithelial damage and inflammatory response in intestinal organoids

**Figure 4:**
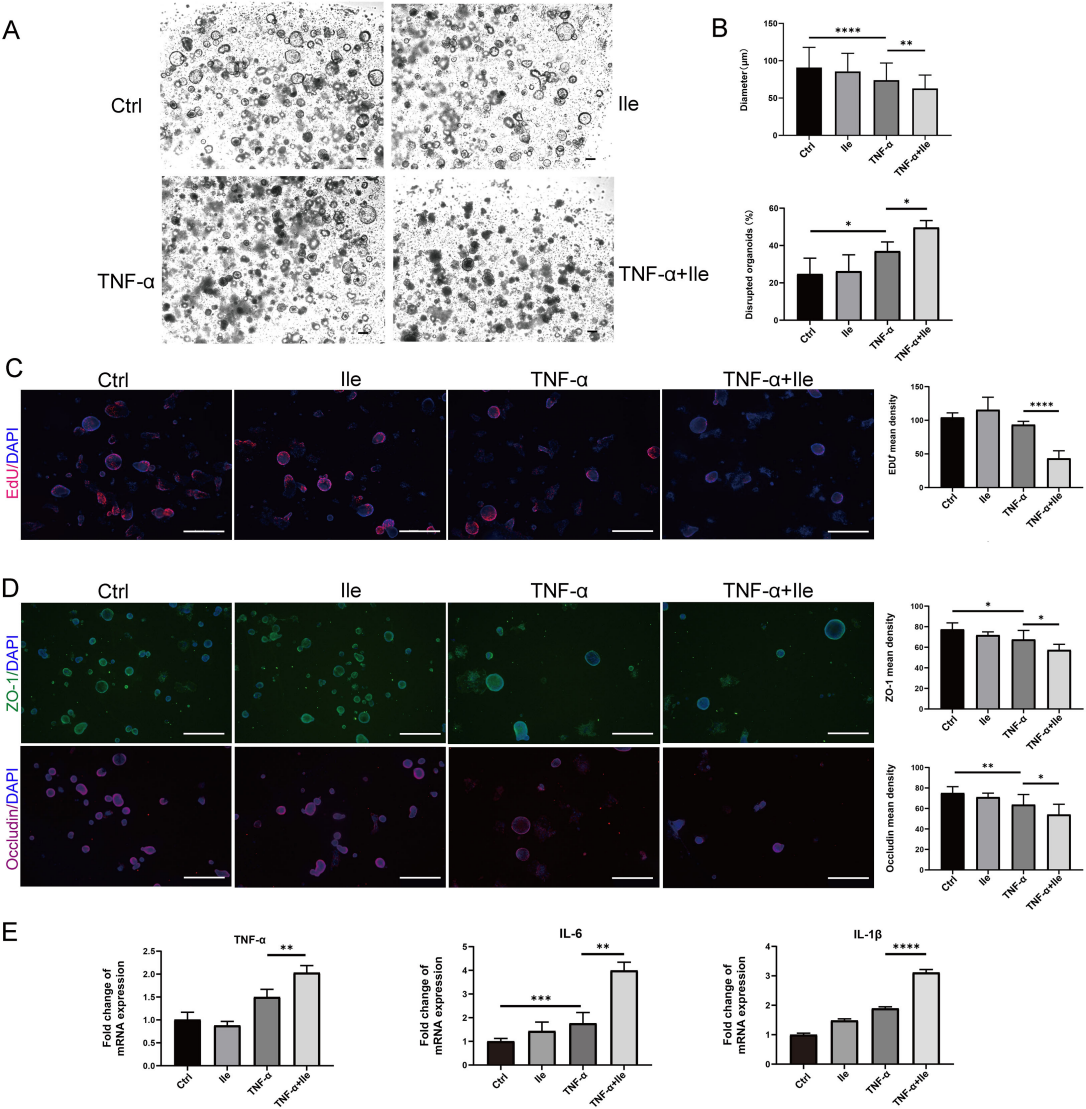
Ile promoted intestinal barrier damage and inflammatory response induced by TNF-α in intestinal organoids. **(A)** Organoids were treated with TNF-α (100 ng/ml) for 24h alone and cocultured with 10 mM Ile for another 24h (scale bars = 100 μm). **(B)** The diameter and the number of damaged organoids were counted; *n* = 100 organoids in each group. **(C)** Organoids were stained with 5-ethynyl-2′-deoxyuridine (EdU) (red). Nuclei were stained with 4′,6-diamidino-2-phenylindole (DAPI) (blue) (scale bars = 500 μm). **(D)** Immunofluorescence for ZO-1 and Occludin in intestinal organoids (scale bars = 500 μm). **(E)** RT-qPCR analysis of the fold induction of pro-inflammatory cytokines (TNF-α, IL-1β and IL-6) in organoids. The treatments are identical to **(A)**. Results are shown as mean ± SD. *p < 0.05, **p < 0.01, ***p < 0.001, ****p < 0.0001 by one-way ANOVA followed by Bonferroni post-hoc test.

We next assessed the effect of Ile on intestinal epithelial function and inflammatory response in intestinal organoids. The growth of organoids upon Ile exposure was not affected under physiological state (**Figures 4A, B**). Under pathological conditions caused by TNF-α, Ile significantly inhibited the growth of organoids and increased the percentage of disrupted organoids (**Figures 4A, B**). Ile also exacerbated the loss of EdU-positive cells caused by TNF-α treatment to reduce the proliferative ability to repair the damaged epithelia (**Figure 4C**). Immunofluorescent staining showed that Ile significantly decreased the expression of TJ proteins (ZO-1 and occludin) in organoids with TNF-α treatment (**Figure 4D**). Recombinant TNF-α alone can induce TNF-α, IL-6, and IL-1β mRNA, whereas Ile alone had no effect. However, Ile significantly potentiated this induction when recombinant TNF-α was present (**Figure 4E**), indicating that Ile enhanced TNF-α-induced NF-κB activation, thereby leading IECs to upregulate pro-inflammatory cytokines in an autocrine manner, establishing a positive-feedback loop that includes TNF-α itself. In summary, Ile worsened intestinal epithelial dysfunction and the inflammation of intestinal organoids in the condition of TNF-α treatment.

### NF-κB signaling pathway mediated intestinal barrier function damage and inflammatory response induced by Ile

**Figure 5:**
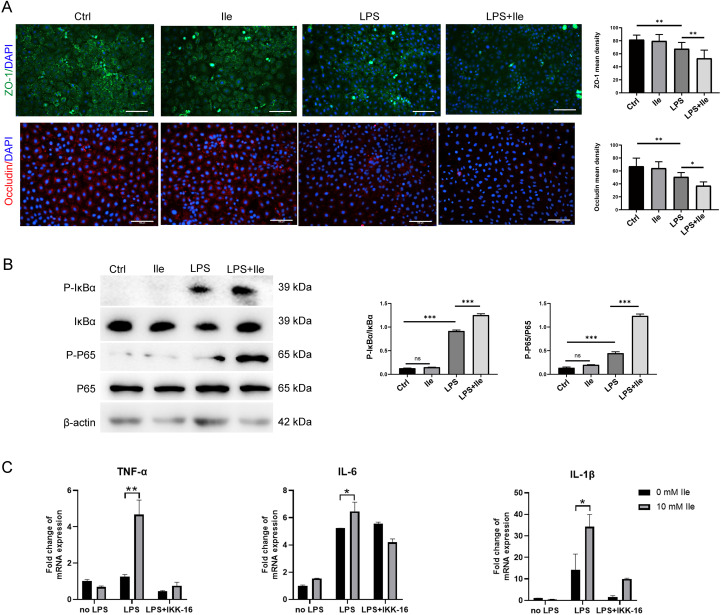
Ile impaired intestinal barrier function and exacerbated inflammatory response by activating NF-κB signaling pathway in IEC-6 cells. **(A)** Immunofluorescence for ZO-1 and Occludin (scale bars = 100 μm). **(B)** Western blot analysis of NF-κB signal pathway. The whole blots are presented in **Supplementary Figure S1**. **(C)** qRT-PCR analysis of relative mRNA levels of pro-inflammatory cytokines (TNF-α, IL-1β, and IL-6) in IEC-6 cells treated with 5 µM IKK inhibition (IKK-16) for 2h, followed by 1 μg/ml lipopolysaccharide (LPS) and 10 mM Ile treatment for two days. Results are shown as mean ± SD. *p < 0.05, **p < 0.01, ***p < 0.001 by one-way ANOVA followed by Bonferroni post-hoc test **(A, B)** or two-tailed Student's *t*-test **(C)**.

The above work showed that the periodontitis-associated metabolite Ile impaired intestinal barrier function and promoted the inflammation *in vivo*. We therefore explored the potential harmful effect of Ile on intestinal epithelial barrier function and inflammatory response *in vitro* using IEC-6 cells. In the presence of LPS, Ile decreased the expression of ZO-1 and occluding (**Figure 5A**), indicating a detrimental effect against LPS-induced impairments on intestinal barrier function. Consistent with the KEGG enrichment analysis above, we found that Ile markedly up-regulated the levels of two NF-κB signaling key molecules, P-P65 and P-IκBα, in IEC-6 cells with LPS induction (**Figure 5B**). Moreover, LPS alone induced TNF-α, IL-6, and IL-1β mRNA expression, whereas Ile alone did not. However, Ile significantly potentiated this induction when LPS was present, and this effect was abolished by the IKK inhibitor IKK-16 (**Figure 5C**). Therefore, these results indicated that Ile impaired intestinal barrier function and exacerbated inflammatory response through activating the NF-κB signaling pathway.

### Inhibiting NF-κB signaling relieved the impairment of intestinal barrier function and inflammation induced by Ile

**Figure 6:**
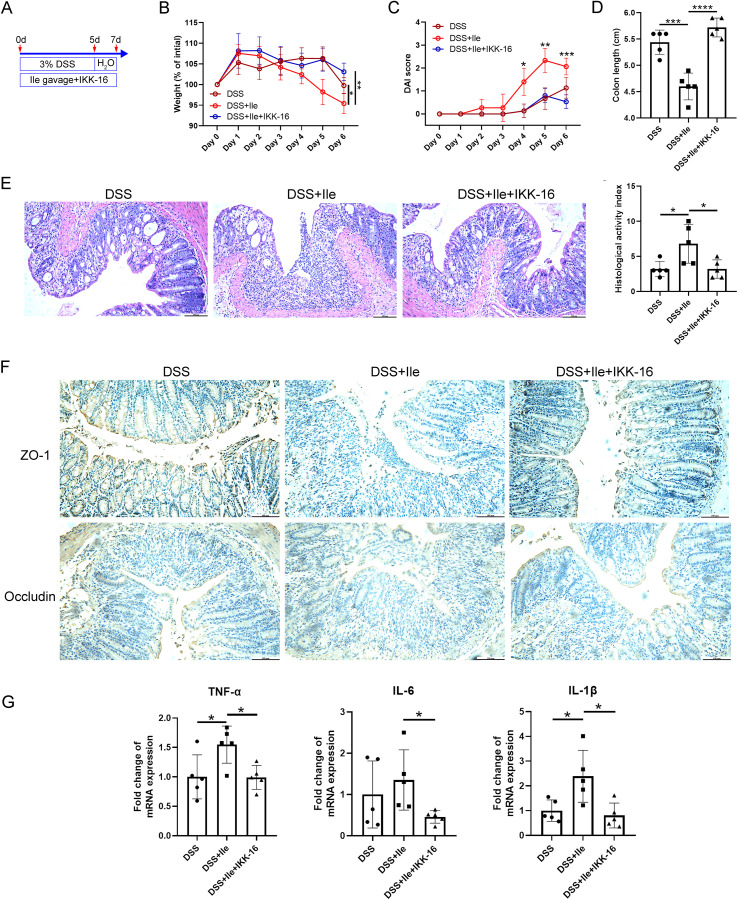
Ile impaired intestinal barrier function and promoted intestinal inflammatory response in colitis mice via NF-κB signaling pathway. **(A)** Schematics of the mouse model. C57BL/6 mice were treated with 3.0% DSS for 5 days, followed by an additional 2 days of regular water in the presence or absence of Ile gavage (50 mg/kg/day) and intraperitoneal injection of IKK-16 (1 mg/kg/day). Body weight **(B)**, DAI score **(C)**, and colon length **(D)** were monitored during the experimental course. **(E)** Representative colonic histological images and the corresponding histological activity index scores (scale bar = 100 μm). **(F)** Immuno-histochemical staining of ZO-1 and Occludin in colonic sections from each group (scale bar = 100 μm). **(G)** qRT-PCR analysis of pro-inflammatory cytokines (TNF-α, IL-1β, and IL-6) mRNA levels in IECs isolated from the colonic tissue in each group. Each dot indicates an individual mouse (*n* = 5). Results are shown as mean ± SD. *p < 0.05, **p < 0.01, ***p < 0.001, ****p < 0.0001 by two-way ANOVA followed by Bonferroni post-hoc test **(A, B)** or one-way ANOVA followed by Bonferroni post-hoc test **(C, D, F)**.

To explore the extent to which the NF-κB signaling pathway induced by Ile was involved in the intestinal barrier disruption and inflammation of colitis, the Ile gavage and DSS-induced colitis mouse model was constructed, and IKK-16 was administered to inhibit the NF-κB signaling pathway. As expected, Ile gavage-DSS mice with IKK-16 treatment displayed rescued body weight, decreased DAI values, higher colon length, and lower colonic HAI scores compared with Ile gavage-DSS mice (**Figures 6A–E**), indicating that the NF-κB signaling pathway played a critical role in Ile aggravating colitis. Immunohistochemical staining of colonic tissue was performed, and the result showed that the expression of TJ proteins (ZO-1 and occludin) in Ile gavage-DSS mice with IKK-16 treatment was significantly increased in contrast to Ile gavage-DSS mice (**Figure 6F**). In addition, the expression of pro-inflammatory cytokines (TNF-α, IL-6, and IL-1β) was significantly decreased in IECs isolated from colonic tissue of Ile gavage-DSS mice with IKK-16 treatment compared with Ile gavage-DSS mice (**Figure 6G**). Collectively, these results suggested that Ile disrupted intestinal barrier function and aggravated intestinal inflammation in colitis mice through the NF-κB signaling pathway.

## Discussion

In response to microbial stimuli or cytokines, NF-κB signaling is activated to impair epithelial barrier function and increase chemokine and cytokine production in IECs (Chen et al., 2021). However, the regulatory mode of NF-κB signaling has not been fully characterized yet. In this study, we found that periodontitis and periodontitis-associated metabolite Ile enhanced NF-κB signaling in IECs to impair epithelial barrier function and increase the expression of pro-inflammatory cytokines (TNF-α, IL-6, and IL-1β) (**Figures 3**–**5**). Therefore, we speculated that periodontitis might disrupt the intestinal barrier and exacerbate intestinal inflammation through its metabolite Ile, which required more work to verify. Therefore, for patients with periodontitis and colitis, more attention should be paid to improving oral health (e.g., removal of pathogenic oral biofilms) to relieve colitis by limiting the levels of pathogenic bacteria and derived metabolites, such as Ile. In addition, reducing dietary Ile intake or targeting Ile catabolism will provide new strategies for the clinical treatment of colitis associated with periodontitis.

UC patients have significantly increased plasma levels of Ile compared with healthy controls (Xin et al., 2015), and the increase of plasma Ile content is positively correlated with the severity of UC (Probert et al., 2018), suggesting that Ile plays a detrimental role in UC. Indeed, Ile gavage administration aggravates the progression of colitis in mice (Wang et al., 2023a). Consistent with these results, we found that Ile impaired intestinal barrier function and exacerbated intestinal inflammatory response by NF-κB signaling (**Figures 3**–**5**). In contrast to these findings, supplementing Ile in the diet for 35 days improves the DSS-induced growth stunting and colonic damage in rats (Mao et al., 2021). We speculate that this might be due to the variations in the species or the methods of animal modeling.

The NF-κB signaling is at the core of the immune responses that also direct aberrant inflammation in the colitogenic gut (Mukherjee et al., 2024). The activation of NF-κB signaling induced the expression of TNF-α, IL-6, and IL-1β, which further amplify the inflammatory response, leading to colonic mucosal damage and persistent inflammation (Luo and Zhang, 2017). Moreover, NF-κB primes the NLRP3-inflammasome for activation by inducing pro-IL-1β and NLRP3 expression, indicating a cross-talk between the NF-κB pathway and the NLRP3 inflammasome (Zhong et al., 2016). Rutaecarpine alleviates gastric mucosal inflammation through suppressing NF-κB pathway and the NLRP3 inflammasome (He et al., 2024). Ile triggers the NLRP3 inflammasome pathway to exacerbate colitis (Wang et al., 2023a). In this study, we found Ile potentiated the NF-κB signaling and up-regulated the expression of TNF-α, IL-6, and IL-1β under inflammatory conditions (**Figures 3**-**5**). Therefore, we hypothesize that NF-κB potentiation by Ile may subsequently prime and potentially enhance the activation of the NLRP3 inflammasome, forming a pro-inflammatory positive feedback loop that aggravates colitis.

Branched-chain amino acids (BCAAs), which include Ile, leucine, and valine, are essential amino acids for animals and humans (Neinast et al., 2019). BCAAs are taken up into cells via BCAA transporters, such as SLC7A5 and SLC43A2 (Kandasamy et al., 2018). It has been shown that BCAAs increase the production of reactive oxygen species (ROS) and stimulate the activation of the NF-κB signaling pathway, favoring the expression of cytokines (TNF-α and IL-6) after uptake by peripheral blood mononuclear cells (Zhenyukh et al., 2017). Consistent with this, we verified that Ile could heighten NF-κB signaling and promote the expression of pro-inflammatory cytokines (TNF-α, IL-6, and IL-1β) *in vivo* and *in vitro* (**Figures 3**-**5**). However, it is still unknown which transporter mediates the uptake of Ile by IECs and whether ROS is involved in the activation of NF-κB signaling by Ile. In the future, more work needs to be done to solve these issues. Notably, Ile alone did not induce TNF-α, IL-6, or IL-1β expression but significantly enhanced their expression in the presence of exogenous TNF-α or LPS (**Figures 4E**, **5C**), suggesting that Ile was a modulatory amplifier of pre-existing NF-κB signaling and cytokine feedback loops rather than an autonomous activator. In addition, the IKK-16 inhibition experiment further supported NF-κB signaling involvement but did not prove the direct effect of Ile. So far, it is unclear how Ile participates in the NF-κB signaling pathway. It is well known that Ile is an activator of mechanistic target of rapamycin (mTOR) complex 1 (mTORC1) activity, which phosphorylates IKKα and IKKβ to activate the NF-κB signaling pathway (Son et al., 2019; Li et al., 2019). Therefore, it was inferred that Ile might enhance the NF-κB signaling pathway through mTORC1.

It is known that compromised intestinal barrier function has a pathogenic role in a number of intestinal and systemic diseases (Odenwald and Turner, 2017). Decreased expression of TJ proteins damages intestinal epithelial barrier function (Ibrahim et al., 2020; Su et al., 2021). Consistent with these studies, we found Ile downregulated the expression of TJ proteins (ZO-1 and occludin) and increased the intestinal permeability (**Figures 2**-**6**). However, how Ile regulates the expression of TJ proteins remains unknown. Numerous studies have shown that activated NF-κB P-P65 moves from the cytoplasm to the nucleus, and then binds to the myosin light chain kinase (MLCK) promoter, initiating the expression of MLCK, which induces the phosphorylation of myosin light chain (MLC) and causes the reallocation of TJ and cytoskeletal proteins, resulting in increased intestinal permeability (Gong et al., 2022; Wang et al., 2021; Ma et al., 2004; Cunningham and Turner, 2012; Shen et al., 2006). In addition, pro-inflammatory cytokines (such as TNF-α and IL-1β) increase the expression of MLCK, which results in decreased expression of TJ protein and thus increased intestinal permeability (He et al., 2020; Lorentz et al., 2017). In this study, NF-κB P-P65 and its downstream pro-inflammatory cytokines (TNF-α, IL-6, and IL-1β) were observably up-regulated by Ile under inflammatory conditions (**Figures 3**-**5**). Therefore, it is supposed that Ile might decrease the expression of TJ proteins through activating the NF-κB/MLCK axis. More studies about this hypothesis are needed to be performed in the future.

In the exploration of the mechanism by which periodontitis impairs the intestinal barrier, the KEGG pathway enrichment analysis showed that IL-17 signaling appeared as highly enriched as NF-κB signaling (**Figure 2C**), indicating a strong transcriptional footprint of the Th17/IL-17 axis in the dual periodontitis-colitis model. Consistent with this result, in 2020, Kitamoto et al. found oral pathobiont-reactive Th17 cells that arose during periodontitis can migrate to the gut and contribute to colitis (Kitamoto et al., 2020b). In addition, we found Ile was an oral pathobiont-synthesizing metabolite that transited from the oral cavity to the gut to aggravate colitis (Wang et al., 2023a). Depletion of Ile had an inhibitory effect on IL-17A production of CD4+ memory T cells (Kang et al., 2024). Therefore, it was speculated that the Th17/IL-17 axis might be an alternate mechanism through which Ile exacerbated colitis.

There are some limitations in our work. First, this study showed that the periodontitis-associated metabolite Ile impaired intestinal barrier function and aggravated intestinal inflammatory response in the context of colitis based on a single-sex study. In the future, these phenotypes should be validated in both sexes. Second, although it has been found that targeting inhibition of NF-κB signaling by IKK-16 can alleviate Ile-induced intestinal barrier dysfunction and inflammation of IECs, the potential long-term toxicity of this inhibitor in humans remains to be further evaluated. In the future, on the one hand, it is necessary to validate these findings in human samples to further explore the mechanism by which Ile enhances the NF-κB signaling pathway and disrupts the intestinal barrier function in periodontitis-mediated colitis. On the other hand, efforts should be made to either accelerate the development of safe and effective NF-κB–targeted therapies or repurpose existing clinically approved NF-κB inhibitors and explore their combination with current colitis treatments to improve the therapeutic effect for patients with periodontitis-mediated colitis. Finally, although our previous study detected elevated Ile levels in saliva and feces of mice with periodontitis, serum Ile remained unchanged (Wang et al., 2023a), suggesting that the oral-gut route may operate independently of systemic circulation. This is consistent with the possibility that locally produced Ile in the oral cavity may be directly transmitted to the gut via swallowing, rather than through systemic absorption and recirculation. The dose of Ile used in the current experiments was higher than the physiological level. However, it remains unclear whether the periodontitis-derived Ile that enters the gut reaches a physiologically relevant concentration sufficient to promote colitis. In the future, the physiological correlation of this route needs to be further explored through metabolic tracing or transport validation.

## Conclusion

Periodontitis-associated metabolite Ile promotes intestinal barrier dysfunction and inflammatory response to aggravate colitis by enhancing NF-κB signaling.

## Data Availability

The data used to support the findings of this study is available from the corresponding author upon request. Requests to access these datasets should be directed to Xueyang Zhang, zxy123@smu.edu.cn.
