# Supplementary material for: Periodontitis-associated metabolite isoleucine impairs intestinal barrier function and exacerbates intestinal inflammatory response by NF-κB signaling
Source: Front Cell Infect Microbiol. 2025 Nov 28;15:1684362. doi: 10.3389/fcimb.2025.1684362 (PMC12698614; doi:10.3389/fcimb.2025.1684362)
Supplement: Supplementary file 1 [file Table1.docx]

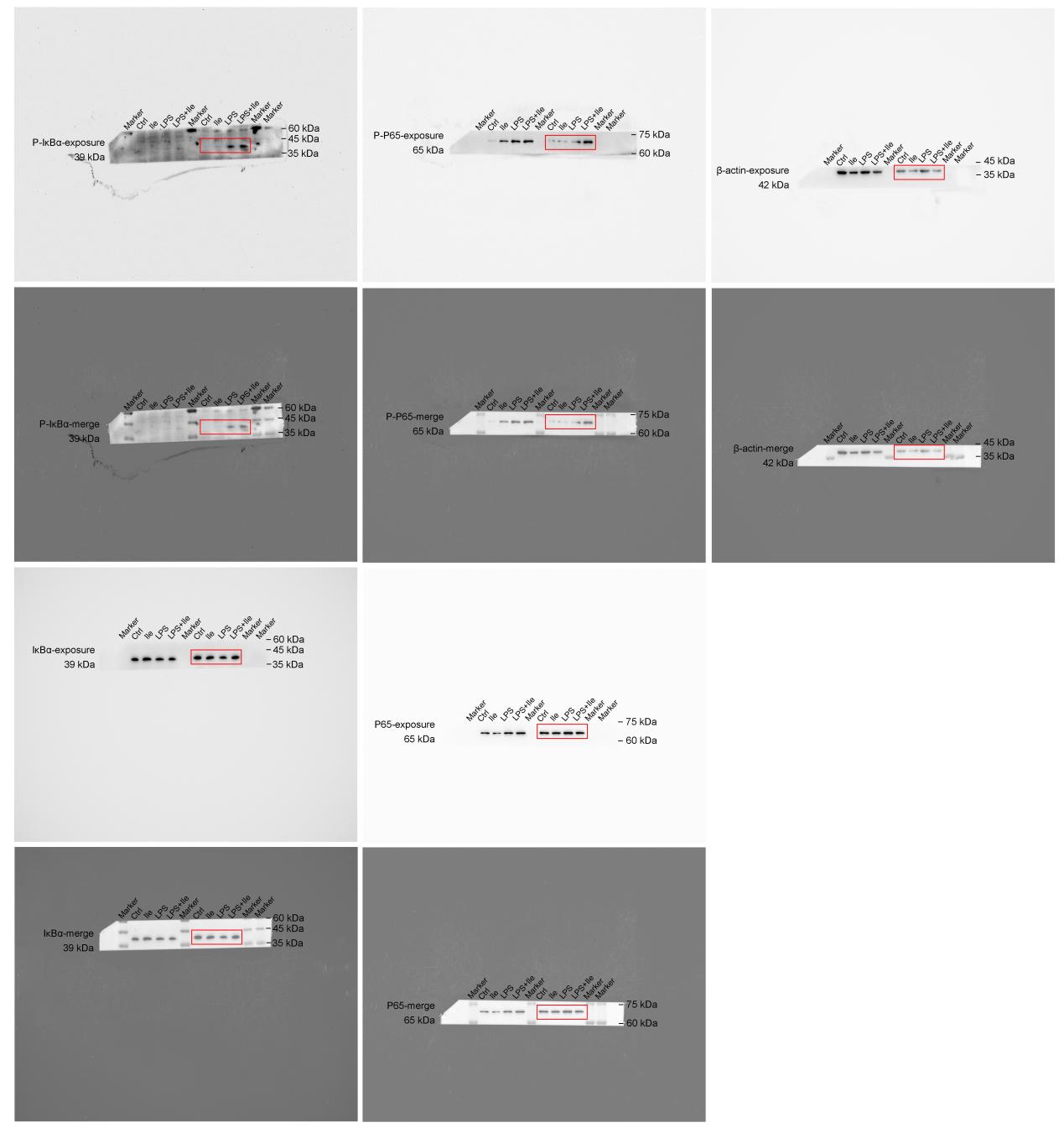


Fig. S1 The original Western blot images for P-IκBα, IκBα, P-P65, P65 and β-actin in IEC-6 cells. The red box represents the blots displayed in the Fig. 5B of manuscript.
